# The International Medical Congress

**Published:** 1867-02

**Authors:** 


### THE INTERNATIONAL MEDICAL CONGRESS.

We have just received the programme adopted by the committee appointed to organize the plan of procedure for the proposed International Medical Congress, to be holden at Paris, in 1867, in connection with the Exhibition, and to which we called the attention of our readers in the issue of the Journal for August. For the information of those who desire to take part in the proceedings, we translate such portions as give the prominent points.

The Congress will be opened on the 16th of August, 1867, under the auspices of the Minister of Public Instruction, and will continue in session two weeks.

It shall be composed of national members or founders, and associate foreign members. The national members will be required to pay each twenty francs, but the associate members are relieved from all pecuniary contribution.

The members of the Congress, national and associate, shall alone take part in the discussions, and the committee have proposed as subjects of discussion the following

I. The anatomy and pathological physiology of tubercle: tuberculization in different countries, and its influence on the general mortality.

II. The common accidents which lead to a fatal result after surgical operations.

III. Is it possible to propose to the different governments any effective measures for restraining the propagation of veneral diseases?

IV. The influence of alimentation employed in the different countries on the production of certain diseases.

V. The influence of climates, races, and different conditions of life in different countries upon menstruation.

VI. The acclimatization of European races in hot countries.

VII. On the entozoa and entophytes which can be developed in man.

Members wishing to make any communication upon the questions of the programme, or to propose any other subject, must send their essays to the General Secretary, at least three weeks before the time of meeting. The committee will decide upon the fitness of the communications, and the order in which they shall be received. Each question will occupy but a single sitting, and a maximum ot twenty minutes only will be allowed for reading each essay.

Accompanying this programme is a commentary on the questions proposed, indicating the points that are especially desired to be brought out in the discussion, and given with the view of securing precision, with the necessary brevity, in the essays. We have no space for it at present, but will endeavor in our subsequent issue to present some of the more important part of the same.—*N.* Y. *Medical Journal.*

